# Transcriptional Profiles of Hybrid *Eucalyptus* Genotypes with Contrasting Lignin Content Reveal That Monolignol Biosynthesis-related Genes Regulate Wood Composition

**DOI:** 10.3389/fpls.2016.00443

**Published:** 2016-04-13

**Authors:** Tomotaka Shinya, Eiji Iwata, Katsuhiko Nakahama, Yujiroh Fukuda, Kazunori Hayashi, Kazuya Nanto, Antonio C. Rosa, Akiyoshi Kawaoka

**Affiliations:** ^1^Agri-Biotechnology Research Laboratory, Nippon Paper Industries Co., Ltd.Tokyo, Japan; ^2^Forest Research Division, Amapa Frorestal e Celulose S.A.Santana, Brazil

**Keywords:** hybrid *Eucalyptus*, lignin, cellulose, flavonoid, transcription factor, RNA-Seq

## Abstract

*Eucalyptus* species constitutes the most widely planted hardwood trees in temperate and subtropical regions. In this study, we compared the transcript levels of genes involved in lignocellulose formation such as cellulose, hemicellulose and lignin biosynthesis in two selected 3-year old hybrid *Eucalyptus* (*Eucalyptus urophylla* × *Eucalyptus grandis*) genotypes (AM063 and AM380) that have different lignin content. AM063 and AM380 had 20.2 and 35.5% of Klason lignin content and 59.0 and 48.2%, α-cellulose contents, respectively. We investigated the correlation between wood properties and transcript levels of wood formation-related genes using RNA-seq with total RNAs extracted from developing xylem tissues at a breast height. Transcript levels of cell wall construction genes such as cellulose synthase (CesA) and sucrose synthase (SUSY) were almost the same in both genotypes. However, AM063 exhibited higher transcript levels of UDP-glucose pyrophosphorylase and xyloglucan endotransglucoxylase than those in AM380. Most monolignol biosynthesis-related isozyme genes showed higher transcript levels in AM380. These results indicate monolignol biosynthesis-related genes may regulate wood composition in *Eucalyptus*. Flavonoids contents were also observed at much higher levels in AM380 as a result of the elevated transcript levels of common phenylpropanoid pathway genes, phenylalanine ammonium lyase, cinnamate-4-hydroxylase (C4H) and 4-coumarate-CoA ligase (4CL). Secondary plant cell wall formation is regulated by many transcription factors. We analyzed genes encoding NAC, WRKY, AP2/ERF, and KNOX transcription factors and found higher transcript levels of these genes in AM380. We also observed increased transcription of some MYB and LIM domain transcription factors in AM380 compared to AM063. All these results show that genes related to monolignol biosynthesis may regulate the wood composition and help maintain the ratio of cellulose and lignin contents in *Eucalyptus* plants.

## Introduction

*Eucalyptus* trees are ubiquitous in tropical/subtropical and temperate zones and are well known for their rapid growth rate and adaptability. The *Eucalyptus* genus is composed of more than 600 species with its origin center based in Oceania (Ladiges et al., 2003). *Eucalyptus* wood is utilized in many ways, such as pulp, paper, civil construction, and furniture and energy production. *Eucalyptus* is likely to be an important bioresource for second generation of biofuels and renewable chemicals in the near future. *Eucalyptus* plantations are increasing around the world. Brazil has more than 600,000 hectares of *Eucalyptus* plantation. Among the various *Eucalyptus* species, the hybrid species (*Eucalyptus urophylla* × *Eucalyptus grandis*) is the most popular one and it grows fast in Brazil. Understanding the mechanism of regulation of the individual components of wood (such as cellulose, hemicellulose, and lignin) is of great commercial importance. The composition of the different components of wood dictates its utility for specific industrial purpose. For example, predominance of cellulose and hemicellulose is optimal for the kraft pulp industry while higher lignin content is preferable for the combustible energy production industry (White, 1987). Several studies have described the structural and regulatory genes involved in secondary cell wall formation (reviewed in Vanholme et al., 2008). Xylem transcription profiles obtained using novel sequencing technologies in three *Eucalyptus* species revealed many genes in wood formation (Salazar et al., 2013).

In particular, genes encoding a cellulose synthase for cellulose biosynthesis were identified in cotton (*Gossypium hirsutum*) (Pear et al., 1996). The Arabidopsis Genome Initiative (2000), provided a reference point for the identification of functional genes in the biosynthetic pathway of several of these components. The *Arabidopsis* genome includes 10 *CesA* gene families (Richmond and Somerville, 2000). The draft genome sequence revealed that *Eucalyptus grandis* has at least 16 *CesA* genes (Myburg et al., 2014). Cellulose and hemicellulose comprise approximately 75% of the cell wall components in woody plants with UDP-glucose as the common precursor (Myburg et al., 2014). Most structural genes of monolignol biosynthesis have been identified in many plant species (**Figure 4A**; Boerjan et al., 2003).

The aromatic lignin polymers commonly found in hardwood plants are primarily composed of two monolignols, the coniferyl and sinapyl alcohols, which form guaiacyl (G) and syringyl (S) lignin when polymerized. This monolignol biosynthesis is carried out via the phenylpropanoid pathway with the conversion of phenylalanine to cinnamate followed by ring hydroxylations, *O-*methylations, and side-chain modifications of various compounds in the pathway. Several lines of evidence emerging from *in vitro* kinetic studies on enzymes, from genetic studies using mutants or transgenic plants with altered expression levels of phenylpropanoid pathway genes, and from studies on metabolites (Humphreys and Chapple, 2002; Boerjan et al., 2003; Vanholme et al., 2013), have provided insight into our current understanding of the lignin biosynthetic pathway. A recent review on this topic suggests that at least 10 enzymes are involved in monolignol biosynthesis (Zhong and Ye, 2015). In the cell wall, PRXs and LACs are believed to catalyze the dehydrogenative polymerization of the monolignols (Boerjan et al., 2003).

**FIGURE 1:**
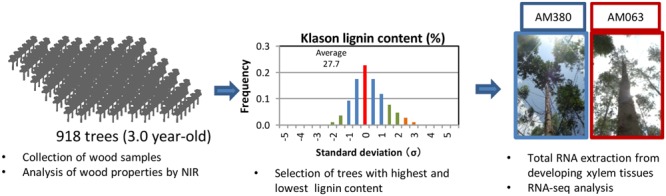
**Workflow: analysis of wood properties and selection of appropriate genotypes.** A total of 918 3-year old elite candidate seed-grown trees were analyzed for wood content and distribution of Klason lignin content. Among these, two genotypes, one with the highest and the other with the lowest Klason lignin content were selected. Total RNAs were extracted from developing xylem tissue in each genotype for further analysis.

In this study, we evaluated wood properties of 918 elite candidate plants using a traditional breeding program, and selected two hybrid *Eucalyptus* genotypes AM380 and AM063, which exhibited the highest (35.5%) and the lowest (20.2%) Klason lignin content at the progeny evaluation stage (**Figure 1**). We then compared the transcript profiles in xylem tissues of these two hybrid genotypes. The progeny evaluation was carried out in seed-grown seedlings generated from parent trees that possessed desirable characters such as good growth rate, basic density or kraft pulp yield. In this study, we focused on the correlation between transcript levels of cellulose, hemicellulose and lignin biosynthesis-related genes, and the wood properties. Flavonoid content and the transcript levels of genes involved in flavonoid biosynthesis were also investigated. We found that several transcription factors such as NAC, AP2/ERF, HD, WRKY, MYB, and LIM acted as positive or negative regulators of lignin biosynthesis. Our results strongly suggest that monolignol biosynthesis-related genes may control wood composition.

## Materials and Methods

### Plant Materials and Wood Samples

**FIGURE 2:**
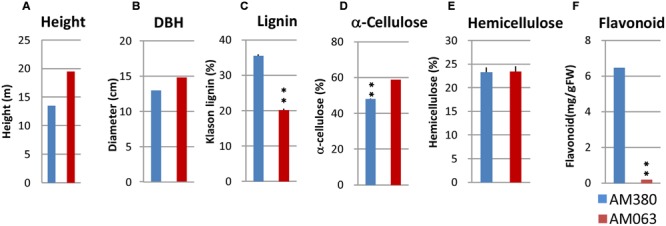
**Growth rate measurement and analysis of wood properties of the two selected genotypes (AM380 and AM063).** Measurements included the height **(A)**, DBH **(B)**, Klason lignin content **(C)**, α-cellulose content **(D)**, hemicellulose content **(E)**, and flavonoid content **(F)**. Wood samples containing developing xylem were air-dried and were ground to a fine powder. NIR spectra were collected for predicting wood chemical composition.

We selected two hybrid *Eucalyptus* genotypes (*Eucalyptus grandis* × *Eucalyptus urophylla*) with contrasting Klason lignin content (**Figure 2C**). These two genotypes were grown in experimental fields (4.97°N, 48.78°W, 44-m elevation) of the plantation company Amapa Frorestal e Celulose S.A., Amapa state, Brazil. Developing xylem tissues were collected for RNA extraction from 3.0 year old individuals of *Eucalyptus urophylla* × *Eucalyptus grandis*.

### RNA Sequencing

The samples of developing xylem were collected by scraping the tissue after removing the bark and immediately treating it with RNA Later (QIAGEN, Hilden Germany) sample solution. We collected three independent RNA samples from each tree. The extracted RNAs were used for preparing the mRNAseq library using the TruSeq RNA sample kit (Illumina, San Diego, CA, USA). High-throughput mRNA sequencing was performed using MiSeq (Illumina, San Diego, CA, USA) and data analysis was carried out using CLC Genomics Workbench (CLC bio, Aarhus, Denmark). *E. grandis* annotation v1.1 in PHYTOZOME v8.0 was used as the reference sequence^1^. Raw read sequences and read counts data have been deposited in DDBJ’s Gene Expression Omnibus and are accessible through series accession number DRA004453.

### Determination of Transcript Levels

The transcript levels of each gene were estimated using the RPKM (RPKMs) value (Garber et al., 2011). Genes of low level RPKM (<10.0) were eliminated. A differentially transcribed gene was defined based on a *t*-test with a 99% confidence rate (cutoff of 0.01).

### Quantitative Reverse Transcription (qRT)-PCR

Quantitative Reverse Transcription-PCR analysis was carried out to validate the transcript levels of selected genes. Total RNAs were separately extracted from the developing xylem tissues from wood samples at a height of 1.2 m from ground level from both genotypes. First strand cDNA was synthesized from 10 μg of total RNA using PrimeScript RT (Perfect Real Time) kit (Takara, Kyoto, Japan). The qRT-PCR analysis was performed as described previously (Shinya et al., 2014). The primers used are listed in **Supplementary Table S5**.

### Chemical Analysis of Cell Wall Composition

Samples for wood properties, Klason lignin, holocellulose, and α-cellulose were collected by using an electric drill (diameter: 1 cm) at breast height (1.2 m). These drill dusts were smashed into a fine powder by Power Mill (Osaka Chemical, Japan) and powders that passed through a #8 mesh were used. Determination of cell wall composition was carried out on dried insoluble cell wall residues (CWR) of samples that were extracted with toluene/ethanol, ethanol, and water using a Soxhlet extractor. Klason lignin was measured using the method described by Eﬄand (1977). Holocellulose was determined by sulfide acid extraction (Wise et al., 1946) and α-cellulose content was measured by the method described by Siddiqui (1976). Hemicellulose content was calculated by reduction of α-cellulose content from holocellulose. Wood samples were measured using at least three replicates.

### Measurement of Wood Properties by NIR

Wood samples containing developing xylem were air-dried for predicting wood chemical composition and were ground to pass through a 1-mm screen. The NIR spectra were collected using NIRFlex (BUCHI, Switzerland) and the NIR (near infrared) spectroscopy was used to evaluate wood chemical components (Inagaki et al., 2009) in *Eucalyptus* (Raymond and Schimleck, 2002).

### Total Flavonoid Content Determination

Wood samples collected as mentioned above were extracted with 80% (v/v) methanol for 1 month at room temperature. Total flavonoid content was determined by aluminum calorimetric method using quercetin as a standard (Quettier-Deleu et al., 2000). Three replicates were used for each test sample.

### Statistical Analysis

Data analysis was performed using Student’s *t*-test using SAS 9.2 software to determine any significant differences between mean values.

## Results

### Analysis of Wood Properties and Selection of Plant Materials

Wood property differences are important for the variations implicated in the anatomical parameters of woods that were investigated. Wood samples at breast height were collected from 918 3-year old *Eucalyptus* trees. Collected wood core samples were grounded by the mill, and basic density, α-cellulose, and Klason lignin were estimated by NIR analysis. Distribution of Klason lignin contents across the 918 trees was between 20.2 and 35.5% and among these, the two genotypes with the highest- (AM380) and the lowest-lignin content (AM063) (**Figures 1** and **2C**) were selected. We also measured the tree height and diameter at breast height (DBH) and calculated the tree volume. The DBH values were almost the same between the two genotypes but the plant height was different with AM063 being considerably taller than AM380 (**Figures 2A,B**). Growth rates of these two selected genotypes were 0.086 m^3^ for AM380 and 0.161 m^3^ for AM063. The two genotypes also exhibited striking difference in their flavonoid content with AM380 showing almost six times higher values than AM063 (**Figure 2F**). Both the flavonoid and Klason lignin content were higher in AM380 and a correlation between the Klason lignin and flavonoid content increase was observed.

### Cellulose and Hemicellulose Biosynthesis

**FIGURE 3:**
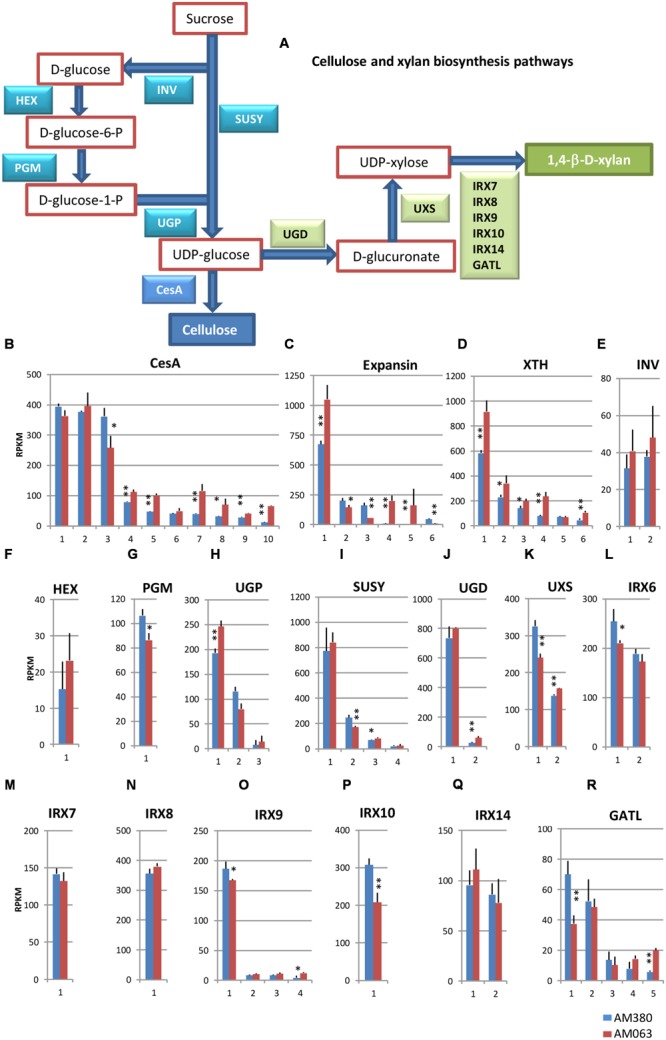
**Cellulose and xylan biosynthesis pathways **(A)** and comparison of the transcript profiles of selected genes related to cell wall construction, CesA **(B)**, Expansin **(C)**, XTH **(D)**, INV **(E)**, HEX **(F)**, PGM **(G)**, UGP **(H)**, SUSY **(I)**, UGD **(J)**, UXS **(K)**, IRX6 **(L)**, IRX7 **(M)**, IRX8 **(N)**, IRX9 **(O)**, IRX10 **(P)**, IRX14 **(Q)**, and GATL **(R)**.** RNAs were extracted from developing xylem tissues of each genotype. Numbers on the X-axis represent gene numbers listed in Table S1. RPKM values are plotted on Y-axis. The data collected from three independent repeats were analyzed. Mean values and standard deviations are shown with error bars. Asterisks or double asterisks indicate significant differences at *p* < 0.05 or *p* < 0.01, respectively.

We investigated the transcript levels of cellulose and hemicellulose biosynthesis pathway-related genes in both the genotypes (**Figure 3A**; **Supplementary Table S1**).

Comparison of the transcript levels of CesA showed that the 10 isozyme genes showed relatively higher transcript levels in the developing xylem tissue (**Figure 3B**). Among them three CesA genes exhibited significantly high transcription, out of which two were at comparable levels between the two genotypes and one (#3) showed higher expression in AM380. Although the other seven CesA genes seemed to have significantly higher transcript levels in AM063, the sum of transcript levels of these 10 CesA genes was almost the same in both genotypes.

Expansins characteristically cause relaxation of wall stress and irreversible cell wall extension. Of the six isozyme genes profiled, three isotypes of expansin showed significantly high expression in AM063 compared to AM380 (**Figure 3C**). Five xyloglucan endotransglucoxylase (XTH) isozyme genes in AM063 showed significantly higher levels than those in AM380 (**Figure 3D**). Four SUSY genes exhibited similar levels of expression in both genotypes (**Figure 3I**). One UGP gene exhibited very high expression and was significantly higher in AM063 than in AM380 (**Figure 3H**). The irregular xylem (IRX) genes are responsible for different functions. For example, IRX6 COBRA encodes extracellular glycosyl-phosphatidyl inositol-anchored protein. The IRX6 gene is expressed at high levels in both the genotypes, with AM380 showing a higher expression than AM063 (**Figure 3L**). IRX9 #1 and IRX10 showed higher transcript levels in AM380 than AM063 (**Figures 3O,P**).

### Lignin Biosynthesis

**FIGURE 4:**
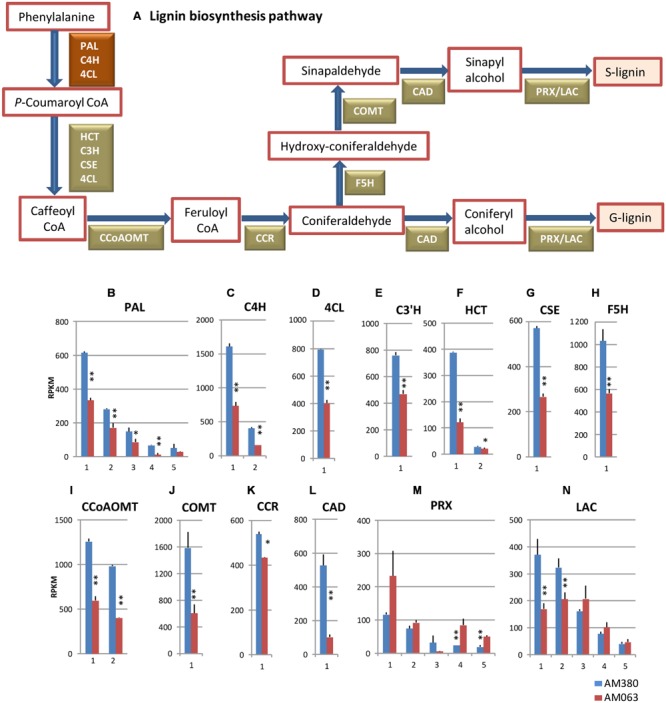
**Lignin biosynthesis pathway (A) and comparison of the transcript profiles of genes related to lignin biosynthesis, PAL (B), C4H (C), 4CL (D), C3′H (E), HCT (F), CSE (G), F5H (H), CCoAOMT (I), COMT (J), CCR (K), CAD (L), PRX (M) and LAC (N).** Numbers on the X-axis represent gene numbers listed in **Supplementary Table S2**. RPKM values are plotted on the Y-axis. Data collected from three independent repeats were analyzed. Mean values and standard deviations are shown with error bars. Asterisks or double asterisks indicate significant differences at *p* < 0.05 or *p* < 0.01, respectively.

The most important metabolic pathway in the developing xylem tissue at breast height is the lignin biosynthesis pathway. We measured the transcript levels of important genes involved in the lignin biosynthesis pathway (**Figure 4**) and compared the levels between AM063 (with low lignin content) and AM380 (with high lignin content).

The first three reaction steps from phenylalanine to *p*-coumaroyl CoA are in the common pathway of general phenylpropanoid biosynthesis including flavonoids etc., carried out by three genes, PAL, C4H, and 4CL as shown in **Figures 4B–D**. The transcript levels of the five PAL genes in AM380 were almost twofold higher as compared to AM063 (**Figure 4B**). The next hydroxylation step from cinnamate to 4-hydroxy-cinnamate is catalyzed by C4H. The *E. grandis* genome has two C4H genes and the transcripts of these two genes in AM380 were also at higher levels as compared with those in AM063 (**Figure 4C**). 4CL is the third enzyme of the general phenylpropanoid pathway, which catalyzes the feeding of CoA thiol esters of 4-coumarate. 4CL1 was strongly transcribed in AM380 with higher lignin content (**Figure 4D**).

The subsequent reactions from *p*-coumaroyl CoA to caffeoyl CoA, (C3′H; CYP98), hydroxycinnamoyl CoA: shikimate HCT and CSE are all catalyzed. *Arabidopsis* has a single C3′H gene (Raes et al., 2003) and only one gene among the four C3′H members is involved in lignin biosynthesis in xylem tissues of *E. grandis*. HCT catalyzes the reactions both immediately preceding and following the insertion of the 3-hydroxy group by C3′H into monolignol precursors (Hoffmann et al., 2003, 2004). CSE has been recently shown to catalyze caffeoyl shikimate to caffeic acid in *Arabidopsis* (Vanholme et al., 2013). *p*-coumaroyl CoA and caffeoyl CoA act as substrates to transfer an acyl group to the acceptor compound skimate, yielding *p*-coumaroyl shikimate, which is used by HCT. C3′H, HCT, and CSE showed significantly strong transcript levels in AM380 (**Figures 4E–G**).

Monolignol biosynthesis pathway has two methylation steps carried out by CCoAOMT and COMT (Ye and Varner, 1995). We observed that two CCoAOMT genes and a COMT gene showed higher transcript levels in AM380 (**Figures 4I,J**). (F5H; CYP84) is involved in the pathway leading to S lignin and catalyzes the step to synthesize hydroxyconiferaldehyde from coniferaldehyde. In the hybrid *Eucalyptus*, there is only one F5H gene expressed in xylem tissue and a strong expression of F5H was observed in AM380 (**Figure 4H**).

CCR catalyzes the first step of monolignol biosynthesis by converting cinnamoyl CoA esters to their corresponding cinnamaldehyde. CAD is involved in one of the final steps of monolignol biosynthesis that catalyzes the reduction of cinnamyl aldehyde to cinnamyl alcohol prior to polymerization into the lignin polymer. The *CCR* and *CAD* genes exhibited higher transcript abundances in AM380 (**Figures 4K,L**).

Class III plant PRXs are secreted plant enzymes that are present in all land plants but absent in unicellular green algae (Passardi et al., 2004). PRXs typically exist as large gene families, with 73 genes present in *Arabidopsis thaliana* (Welinder et al., 2002) and 138 in rice (*Oryza sativa*) (Passardi et al., 2004). In *Eucalyptus grandis* there are 45 class III *PRX* genes and 23 LAC genes^2^ (EucaGenIE). Monolignol polymerizing enzymes PRX and LAC showed varying abundance of transcripts between the two genotypes. Most of the PRX transcripts of AM380 exhibited lower levels of expression compared to those of AM063, while two major LAC genes in AM380 showed higher transcript levels (**Figures 4M,N**).

### Flavonoid Biosynthesis Pathway

**FIGURE 5:**
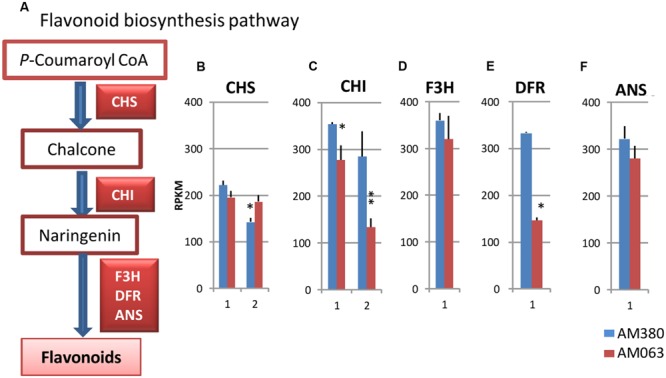
**Flavonoid biosynthesis pathway **(A)** and transcript profiles of genes related to flavonoid biosynthesis, CHS **(B)**, CHI **(C)**, F3H **(D)**, DFR **(E)** and ANS **(F)**.** Numbers on the X-axis represent gene numbers listed in **Supplementary Table S2**. RPKM values are plotted on the Y-axis. Data collected from three independent repeats were analyzed. Mean values and standard deviations are shown with error bars. Asterisks or double asterisks indicate significant differences at *p* < 0.05 or *p* < 0.01, respectively.

The biosynthetic pathways leading to lignin and flavonoids diverge at the common intermediate *p*-coumaroyl CoA (**Figure 5A**). On the flavanoid biosynthesis pathway, the catalysis of the enzyme CHS serves as the initial step. Following the reaction steps, CHI, F3H, DFR and ANS catalyze the various steps leading to the synthesis of flavonoids. In the hybrid *Eucalyptus*, two isogenes of CHS and CHI, a single gene of F3H, DFR and ANS were strongly transcribed in the xylem tissue (**Figures 5B–F**). While CHI and DFR showed higher transcript levels in AM380, the remaining genes showed comparable expression levels between the two genotypes (except for one isogene for CHS; **Figures 5B–F**). Flavonoids are important plant secondary metabolites and are serve as natural regulators of cellular auxin eﬄux and of auxin polar transport (Besseau et al., 2007). Auxins have a crucial role in wood formation (Nilsson et al., 2008). Flavonoid contents in both the *Eucalyptus* genotypes were quite different with AM380 showing much higher flavonoid levels than AM063 (**Figure 2F**).

### Transcription Factors

**FIGURE 6:**
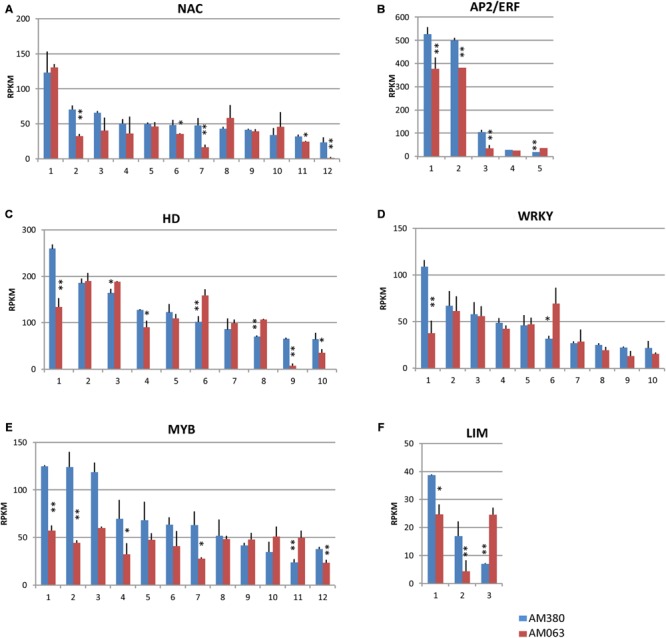
**Comparison of the transcript profiles of transcription factor genes related to cell wall construction, NAC **(A)**, AP2/ERF **(B)**, HD **(C)**, WRKY **(D)**, MYB **(E)**, and LIM **(F)**.** Numbers on the X-axis represent gene numbers and *Arabidopsis* homolog gene names listed in **Supplementary Table S3**. RPKM values are plotted on the Y-axis. The data collected from three independent repeats were analyzed. Mean values and standard deviations are shown with error bars. Asterisks or double asterisks indicate significant differences at *p* < 0.05 or *p* < 0.01, respectively.

Several transcription factors involved in wood formation, including those in lignin biosynthesis such as NAC domain, AP2/ERF, HD, WRKY, MYB, and LIM transcription factors, have been reported (Hussey et al., 2013). Many of their isogenes are transcribed in xylem tissues and we measured the transcript levels in 12 NAC, five AP2/ERF, 10 HD, 10 WRKY, 12 MYB, and 3 LIM transcription factors in the present study (**Figure 6**; **Supplementary Table S3**).

Among the 12 NAC domain transcription factors, four genes (#2, 6, 7, 11, and 12) were strongly transcribed in AM380 (**Figure 6A**). An ethylene response element binding factor-like gene, AP2/ERF class transcription factor, is a large family of genes that is implicated in the abiotic stress response (Mizoi et al., 2012). Two strongly transcribed AP2/ERF genes (#1 and 2) were identified in *Eucalyptus* and both showed significantly high expression levels in AM380 (**Figure 6B**).

Of the 10 HD genes, the transcript levels of #1 (HD-Zip), #4 (HAT22), #9 (KNOX/ELK-HD), and #10 (duplicated HD) were significantly higher in AM380 as compared to AM063 (**Figure 6C**). Most WRKY genes showed no difference between the two genotypes, although one WRKY gene (#1) showed significantly high transcript level of AM380 and one (#6) showed significantly lower levels of expression in AM380 (**Figure 6D**).

MYB transcription factor family is one of the largest protein families. Among the 12 MYB family genes listed, most genes showed higher expression in AM380, indicating a positive regulation of these genes in lignin biosynthesis (**Figure 6E**). Two LIM genes (#1 and #2) showed higher transcript levels in AM380, indicating LIM may promote lignification (**Figure 6F**).

### Reference Genes

We investigated four genes, ubiquitin, tubulin, histone, and actin as reference genes. Ubiquitins are associated with developmental plasticity and environmental adaptation and play a role in several facets of plant growth and development (Cassan-Wang et al., 2012). In addition, these proteins have also been found to be involved in the regulation of xylem differentiation, thereby allowing cells to initiate and progress through the stages of lignocellulose formation. The transcript levels of 10 ubiquitin, 10 tubulin, 8 histone, and 8 actin genes were measured (**Supplementary Table S4**). With the exception of a few, most transcripts showed similar abundances between the two genotypes (**Supplementary Figure S1**). Therefore, the overall physiological conditions in AM063 and AM380 genotypes might not be different with respect to environmental stresses.

### Validation of Transcript Levels by qRT-PCR

To validate the transcript levels by RNA-seq, we measured relative expression levels of several genes, such as CesA2, CesA3, 4CL1, HCT1, CSE, F5H, CHS1, HD1, MYB2, and LIM1 by qRT-PCR with UBI1 gene as a reference gene. The relative transcript levels of these ten genes obtained from qRT-PCR experiments exhibited similar values to those obtained from RNA-seq experiments (**Supplementary Figure S2**). This confirmed the reliability of the RNA-seq data obtained in this study.

## Discussion

In this study, we compared the transcript levels of several secondary cell wall formation-related genes in developing xylem tissues of two genotypes (AM380 and AM063) with extremely different lignin and α-cellulose contents. Klason lignin content of the 918 3 year-old field-grown genotypes showed a normal distribution (**Figure 1**). A previous study has reported negative correlation between plant growth and lignin content in 396 populous genotypes (Novaes et al., 2010). Transgenic populous trees with antisense 4CL showed reduced lignin, compensated cellulose and enhanced growth in a greenhouse (Hu et al., 1999). We did not observe a correlation between the growth rates and Klason lignin contents in this study (data not shown). The two selected genotypes had extremely different Klason lignin content, but the α-cellulose content of each genotype seems to compensate for the difference in Klason lignin, and the hemicellulose contents were almost the same in both genotypes (**Figure 2**).

The analysis of transcripts data of the genes involved in lignin biosynthesis showed that the common phenylpropanoid genes and monolignol biosynthesis genes displayed overall higher transcript levels in AM380 (**Figure 4**). Higher transcript levels of PAL, C4H, and 4CL, the first three genes in the pathway, were observed in AM380 compared to AM063. The elevated transcript levels observed in most genes in AM380 (**Figure 4**) could explain the high Klason lignin contents in this genotype. In contrast to the monolignol biosynthesis genes, the genes involved in flavonoid biosynthesis, such as CHS, F3H, and ANS, showed almost similar transcript levels among the two genotypes, with the exception of two CHI and one DFR genes, which exhibited higher transcript levels in AM380 (**Figure 5**). This may indicate that CHI and DFR are possibly the key enzymes in flavonoid biosynthesis and these two genes may be crucial for this pathway. In fact, CHI has been shown to enhance flavonoid production and flower pigmentation in Japanese morning glory (Morita et al., 2014). DFR is reported to be a key regulatory gene for pigmentation in crabapples (Tian et al., 2015).

Flavonoids are important plant secondary metabolites serving various functions in higher plants, including pigmentation, UV protection, fertility, antimicrobial defense, and the recruitment of nitrogen-fixing bacteria (Cain et al., 1997). Flavonoids are also known to act as auxin transport inhibitors (Besseau et al., 2007). As auxins have a crucial role in wood formation, higher flavonoid content results in the obstruction of wood formation. The low growth rate observed in AM380 (**Figure 2**) may have been caused by the higher flavonoid content. A previous study has reported a negative correlation between wood formation and lignin content in woody plants (Novaes et al., 2010). However, detailed analysis of correlations between growth rates and flavonoid or lignin contents has not been carried out until now. Our results showed no correlation between the transcript abundances of HCTs and CHSs, suggesting that HCT and CHS gene expressions are independently regulated. This observation is supported by previous transcript analysis studies in young *E. globulus* plants (Shinya et al., 2014).

The transcript levels of the cellulose biosynthesis pathway genes CesA and SUSY were not different between these two genotypes. However, other genes, such as INV, HEX, and UGP, transcribed at higher levels in AM063 compared to AM380 (**Figure 3**). In the 1,4-β-D-xylan biosynthesis pathway, transcript levels of most genes were similar, except for UXS, IRX10, and GATL. The analysis of wood properties revealed that hemicellulose contents were similar in both genotypes (**Figure 2**), suggesting that the hemicellulose content may be regulated independently. A previous report has shown that the transgenic poplar plants with antisense 4CL showed compensated relationships between cellulose and lignin contents (Hu et al., 1999).

In the monolignol biosynthesis pathway, most genes exhibited significantly higher levels of transcription in AM380 than in AM063 (**Figure 4**). In particular, the two CCoAOMT genes and a CCOMT were strongly expressed in the xylem tissue and were obviously at much higher levels in AM380 (**Figures 4I,J**). CCoAOMT catalyzes the methylation of cafferoyl CoA to feruloyl CoA. Several reports on the functional analysis of CCoAOMT suggest that this enzyme likely catalyzes the G unit biosynthesis. COMT is an *O*-methyltransferase that tends to be broad in substrate affinity and can potentially act in various branches of the phenylpropanoid pathway. The highly conserved S-adenosyl methionine (SAM) binding domain in COMT proteins indicates the use of SAM for the methylation of hydroxyl-coniferaldehyde to sinapaldehyde (Davin et al., 2008). In our study, strong transcript levels (RPKM: >400) were observed in all genes of monolignol biosynthesis in AM380 (**Figure 4**). In general, the transgenic downregulation of CAD does not affect the total lignin content. The inhibition of monolignol biosynthesis results in changes in lignin composition, such as incorporation of accumulated aldehyde precursors or novel units into the lignin polymer, the kind of changes that would render biomass more digestible (Li et al., 2008). However, transgenic research in several plant species with downregulation of other lignin biosynthetic genes seems to indicate decreased lignin content in these plants (Li et al., 2008).

Transcript levels of the monolignol polymerizing enzyme genes, such as PRX and LAC, showed contrasting results. RNA-seq data did not indicate any monolignol-specific PRX genes as we did not observe higher expression of this gene in AM380 (**Figure 4M**). On the other hand, two LAC genes were transcribed at significantly higher levels in AM380 than those in AM063. Class III PRX enzymes have a broad substrate specificity (Kawaoka et al., 2003). Even though there is apparent functional redundancy, the cellular localization and functions of most of the isoenzymes coded by different *PRX* genes remain poorly understood. A study on the *Arabidopsis thaliana lac4* and *lac17* double knockout mutant provided the first *in vivo* evidence that these two LAC genes are involved in monolignol polymerization (Berthet et al., 2011). In this report, the disruption of both the LAC genes led to a small decrease in lignin content and the deposition of S lignin units was virtually unchanged in the mutant, suggesting the presence of additional lignin LAC genes or alternate polymerization enzyme in *Arabidopsis*. Similar assumption can be made for *Eucalyptus* sp. as well.

Several transcription factors, such as NAC, AP2/ERF, HD, WRKY, MYB, and LIM, act as positive or negative regulators of lignification. In *Arabidopsis*, SND1, NST1, NST2, VND6, and VND7 are considered as primary regulators of secondary cell wall deposition (Zhong and Ye, 2015). The NAC #1 and #3 were similar to AtSND1 and AtNST1, respectively, at the amino acid sequence level, and these genes are possibly orthologous genes. An ethylene response element binding factor-like gene, AP2/ERF class transcription factor is a large family that is implicated in the abiotic stress response (Mizoi et al., 2012). This gene is differentially expressed and its expression is localized in the phloem in the secondary tissue of aspen. In hybrid *Eucalyptus*, two strongly transcribed AP2/ERF genes were observed (**Figure 6B**). The HD-containing superfamily of transcription factors participate in a wide variety of plant developmental processes. Transcription factors, such as the leucine zipper-associated HD-Zip, wushel-related WOX, knotted-related KNOX, and zinc finger-associated ZF-HD homeodomain containing transcription factors, have been associated with various processes related to meristem function, organ polarity, and vascular development in several species (Ariel et al., 2007). In the hybrid *Eucalyptus*, Homeobox-leucine zipper protein, wuschel-related homeobox protein, and knotted-related KNOX protein were highly expressed in developing xylem (**Supplementary Table S3**). The WRKY transcription factors are known to be upregulated in *Arabidopsis* stem secondary growth and xylem tissue (Ko et al., 2004). Mutants in WRKY transcription factors are reported to be associated with increased stem biomass (Wang et al., 2010). In our study, several WRKY genes exhibited high levels of transcription in the *Eucalyptus* genotypes (**Figure 6D**).

Several MYB transcription factors have been shown to be important regulators of secondary cell wall formation. Goicoechea et al. (2005) have previously shown that the *Eucalyptus gunnii* EgMYB2 protein binds to the CCR and EgCAD2 gene promoters and activates their transcription. The constitutive overexpression of *AtMYB46*, the putative *EgMYB2* orthologue in *Arabidopsis*, has been shown to be associated with ectopic lignification, secondary cell wall thickening and activation of lignin and other related genes (Ko et al., 2009). In the present study, high RPKM values were observed in the MYB transcription factor positive regulator candidate genes (#1, 2, 3, and 12) and the negative regulator candidate gene (#11) (**Figure 6E**).

Another transcription factor, the LIM domain transcription factor, binds specifically to a PAL-box element, which is thought to be an important *cis*-acting element of lignification-related gene expression (Kawaoka et al., 2000; Kaothien et al., 2002). Transgenic tobacco and *E. camaldulensis* plants with antisense LIM show low concentrations of transcripts of some key genes such as *PAL* and *4CL* that are involved in the lignin biosynthesis pathway (Kawaoka et al., 2000, 2006; Negishi et al., 2011). Thus, LIM seems to play a crucial role in lignification. Recently, cotton LIM protein has been shown to have dual functions in the promotion of biosynthesis of the lignin or lignin-like phenolics in secondary wall and in the mediation of crosstalk between the cytoplasm and nucleus in developing fibers (Han et al., 2013). In the present study, one of the LIM genes (#3) showed lower transcript levels in AM380, and may act as a negative regulator for lignification (**Figure 6F**).

In this study we investigated the transcript levels of many genes involved in secondary cell wall formation in two hybrid *Eucalyptus* plants with contrasting lignin content. From the results of RNA levels and analysis of wood properties, it is apparent that genes related to monolignol biosynthesis may regulate the wood composition and help maintain the ratio of cellulose and lignin contents. Methods that aid in effective biomass production have gained considerable ecological significance in recent years with the multifarious uses of the biomass in various industries. Selection of elite trees is important for genome-wide association studies as well. *Eucalyptus* plantation and breeding companies are constantly working on effective selection of superior trees that have higher growth rates and cellulose fiber yield. Our results indicate that lower transcript abundances of monolignol biosynthesis related genes could provide useful selection markers for lower lignin contents. Our results provide a strong dataset for selecting elite candidate trees through monitoring the transcript levels of several useful genes.

## Conclusion

We investigated the correlation between transcript levels of cellulose, hemicellulose, and lignin biosynthesis-related genes and wood composition using two hybrid *Eucalyptus* genotypes with contrasting Klason lignin contents. These two genotypes had same amount of hemicellulose, and compensated cellulose and lignin contents. Transcript levels of cellulose and hemicellulose biosynthesis genes did not exhibit remarkable correlation with cellulose and hemicellulose contents except for UGP. However, most monolignol biosynthesis-related genes exhibited higher transcription levels in AM380, the genotype with higher lignin content. Therefore, these results suggest that genes related to monolignol biosynthesis may regulate the composition of wood in *Eucalyptus* plants.

## Author Contributions

TS performed the experiments and analyzed the data, and drafted the manuscript. EI, YF, KH, and KNan contributed selection and collection of samples, and analyzed wood properties, and flavonoid contents. KNak analyzed transcript levels by qRT-PCR. AR analyzed wood properties’ data. AK conceived the idea, designed the study and in interpreting results and made the manuscript. All authors read and approved the final manuscript.

## Conflict of Interest Statement

The authors declare that the research was conducted in the absence of any commercial or financial relationships that could be construed as a potential conflict of interest.

The reviewer HW and handling Editor declared their shared affiliation, and the handling Editor states that the process nevertheless met the standards of a fair and objective review.

## Footnotes

1 http://www.phytozome.net/eucalyptus.php

2 http://eucgenie.org/

## Supplementary Material

FIGURE S1 **Transcript profiles of reference genes, ubiquitin (A), tubulin (B), histone (C) and actin (D).** Numbers on the X-axis represent gene numbers listed in **Table S4**. RPKM values are plotted on the Y-axis. Asterisks or double asterisks indicate significant difference at *p* < 0.05 or *p* < 0.01, respectively.

FIGURE S2 **Relative transcript levels of CesA2, CesA3, 4CL1, HCT1, CSE, F5H, CHS1, HD1, MYB2 and LIM1 genes in the two genotypes measured by quantitative real-time PCR analysis.** UBI1 gene was used as the reference gene. The error bars represent SD values (*n* = 3). Asterisks or double asterisks indicate significant difference at *p* < 0.05 or *p* < 0.01, respectively.

TABLE S1 **List of cellulose and xylan biosynthesis pathway genes and mean values of their transcript levels in both genotypes**.

TABLE S2 **List of lignin and flavonoid biosynthesis pathway genes and mean values of their transcript levels in both genotypes**.

TABLE S3 **List of transcription factors and mean values of their transcript levels in both genotypes. *Arabidopsis* homolog names are also listed**.

TABLE S4 **List of reference genes and mean values of their transcript levels in both genotypes**.

TABLE S5 **Gene-specific primers used in quantitative RT-PCR**.

## Abbreviations

ANS: anthocyanidin synthase
CAD: cinnamyl alcohol dehydrogenase
CCoAOMT: caffeoyl-CoA 3-O-methyltransferase
CCR: cinnamoyl-CoA reductase
CesA: cellulose synthase
C3H: coumaroyl-CoA 3-hydroxylase
C4H: cinnamate-4-hydroxylase
CHI: chalcone isomerase
CHS: chalcone synthase
4CL: 4-coumarate-CoA ligase
COMT: caffeic acid:5-hydroxyferulic acid O-methyltransferase
CSE: caffeoyl shikimate esterase
DFR: dihydroflavonol 4-reductase
F3H: flavonone 3-hydroxylase
F5H: ferulate 5-hydroxylase
GATL: galaxturonosyl transferase-like
HCT: hydroxycinnamoyl-CoA shikimate/quinate hydroxycinnamoyl transferase
HD: homeodomain
HEX: hexokinase
INV: invertase
LAC: laccase
PAL: phenylalanine ammonium lyase
PGM: phosphoglucomutase
PRX: peroxidase
RPKM: reads per kilobase of exon per million fragments
SUSY: sucrose synthase
UGD: UDP-glucose dehydrogenase
UGP: UDP-glucose pyrophosphorylase
UXS: UDP-xylose synthase
