# Supplementary material for: Transcriptional Profiles of Hybrid Eucalyptus Genotypes with Contrasting Lignin Content Reveal That Monolignol Biosynthesis-related Genes Regulate Wood Composition
Source: Front Plant Sci. 2016 Apr 13;7:443. doi: 10.3389/fpls.2016.00443 (PMC4829581; doi:10.3389/fpls.2016.00443)
Supplement: FIGURE S1 — Transcript profiles of reference genes, ubiquitin (A), tubulin (B), histone (C) and actin (D). Numbers on the X-axis represent gene numbers listed in Table S4. RPKM values are plotted on the Y-axis. Asterisks or double asterisks indicate significant difference at p < 0.05 or p < 0.01, respectively. [file Image_1.PDF]

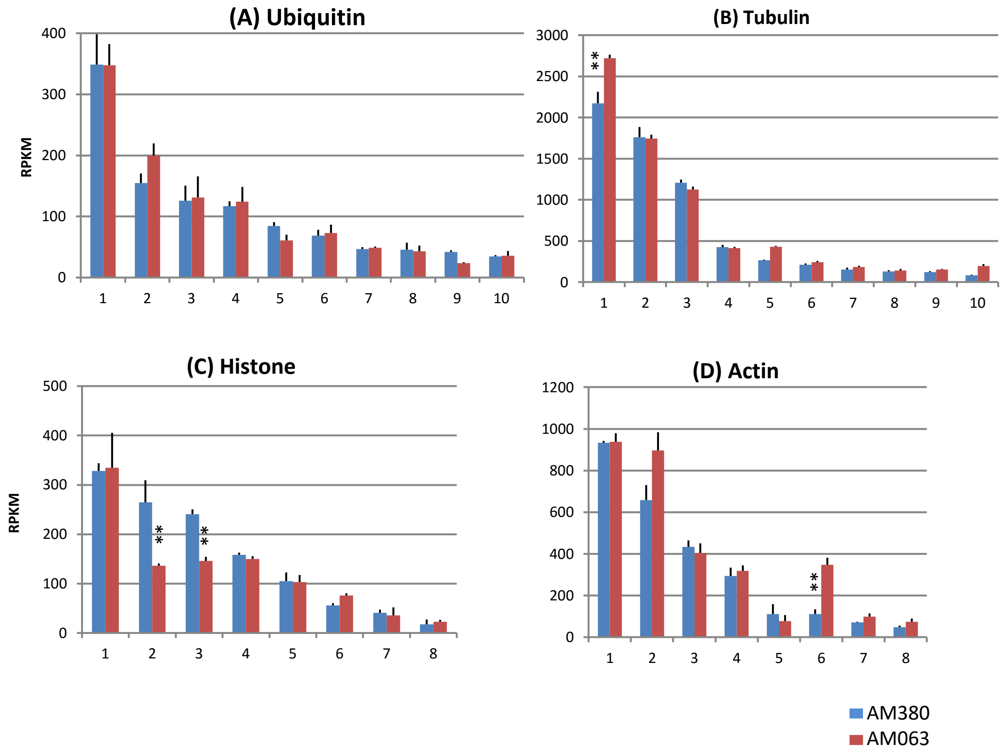

Supplementary Figure S1

Transcript profiles of reference genes, ubiquitin (A), tubulin (B), histone (C) and actin (D). Numbers on the X-axis represent gene numbers listed in Table S4. RPKM values are plotted on the Y-axis. Asterisks or double asterisks indicate significant difference at $p<0.05$ or $p<0.01$, respectively.
