# Supplementary material for: Transcriptional Profiles of Hybrid Eucalyptus Genotypes with Contrasting Lignin Content Reveal That Monolignol Biosynthesis-related Genes Regulate Wood Composition
Source: Front Plant Sci. 2016 Apr 13;7:443. doi: 10.3389/fpls.2016.00443 (PMC4829581; doi:10.3389/fpls.2016.00443)
Supplement: Supplementary file 5 [file Presentation_2.PDF]

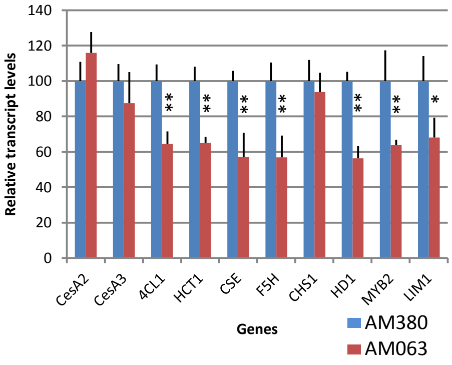

Supplementary Figure S2
Relative transcript levels of CesA2, CesA3, 4CL1, HCT1, CSE, F5H, CHS1, HD1, MYB2 and LIM1 genes in the two genotypes measured by quantitative real-time PCR analysis. UBI1 gene was used as the reference gene. The error bars represent SD values (n=3). Asterisks or double asterisks indicate significant difference at p<0.05 or p<0.01, respectively.
